# SIRT7 Downregulation Promotes Breast Cancer Metastasis Via LAP2α-Induced Chromosomal Instability

**DOI:** 10.7150/ijbs.75340

**Published:** 2023-03-05

**Authors:** Qin Huo, Siqi Chen, Jialang Zhuang, Chuntao Quan, Yue Wang, Ni Xie

**Affiliations:** Biobank, Department of Laboratory Medicine, Shenzhen Institute of Translational Medicine, Shenzhen Second People's Hospital, First Affiliated Hospital of Shenzhen University, Health Science Center, Shenzhen University, Shenzhen, 518035, China.

**Keywords:** SIRT7, chromosomal instability, LAP2α, breast cancer, metastasis

## Abstract

Chromosomal instability (CIN) plays an important role in the initiation and progression of carcinomas. However, the regulatory mechanism of metastasis mediated by CIN in breast cancer is not fully understood. Here, we aimed to demonstrate that the deregulation of SIRT7 and lamina-associated polypeptide 2α (LAP2α) critically contributes to CIN-induced metastasis in breast cancer. Expression of SIRT7 and chromosome stability-related genes was examined using western blotting, quantitative real-time PCR, immunohistochemistry, and immunofluorescence; functional significance of SIRT7 was examined using *in vitro* and *in vivo* models; and interaction between SIRT7 and LAP2α was assessed by co-inmunoprecipitation (Co-IP) assays. Doxorubicin (DOX) inhibited SIRT7 expression and enhanced CIN in breast cancer cells; SIRT7 deficiency led to CIN in breast cancer cells. Co-IP approach and immunohistochemistry demonstrated that SIRT7 interacted directly and positively with LAP2α and SIRT7 knockdown led to increased ubiquitination-dependent degradation of LAP2α and reduced protein levels of LAP2α, whereas LAP2α knockdown did not affect SIRT7 expression. *In vitro* and *in vivo* evidence revealed that SIRT7 promotes breast cancer metastasis through the SIRT7/LAP2α axis. In summary, SIRT7 interacts with LAP2α to regulate CIN and metastasis in breast cancer, and inhibition of SIRT7/LAP2α axis represents a potential therapeutic strategy for preventing breast cancer metastasis.

## Introduction

Chromosomal instability (CIN), characterized by a high rate of structural and quantitative chromosomal aberrations, is the main driver of tumor evolution 1, 2. The causes of CIN include spindle checkpoints dysfunction, centrosome replication disorders, kinetochore-microtubule attachment, mitotic DNA damage, and replication stress 3, 4. Intracytoplasmic micronuclei are considered important markers of CIN and chromatin or DNA damage. Increased frequency of micronucleated cells is an indicator of chromatin damage and apoptosis 5, 6. Cytogenetic analysis of more than 60,000 human cancer samples shows that most solid tumors, and their metastases are associated with chromosomal aberrations 7. Previous studies have shown that when the incidence of CIN is too high or too low, the clonal fitness of cells decreases 8, 9, and moderate CIN alone diversify tumor populations, retain favorable genotypes, and promote tumor cell proliferation and genome evolution, and moderate CIN is associated with tumor therapy resistance and an increased risk of death 10, 11; However, the contribution of CIN to tumor metastasis remains unclear.

Mammalian lamina-associated polypeptide (LAP) 2α, encoded by the thymopoietin, is a member of the LAP2 family 12. LAP2α may regulate nuclear structure maintenance, assembly, positioning of the mitotic spindle, DNA repair, and gene transcription by interacting with Emerin, Lamin A/C, or BAF1 13. Importantly, LAP2α participates in telomere maintenance or protection by associating with telomere repeat binding factor 1 (TRF1) and Werner syndrome helicase (WRN) 14, 15. Pekovic et al. demonstrated that LAP2α promotes the proliferation of human diploid fibroblasts by controlling the intranuclear sublocalization and phosphorylation levels of retinoblastoma protein (Rb). These studies suggest that LAP2α is an important molecule that regulates chromatin stability and is associated with cancer progression 16.

SIRT7, a member of the SIRT family of NAD+ -dependent protein deacetylases, is a key mediator of several cellular activities. Its important role in tumorigenesis and development has received increasing attention 17. Elevated levels of SIRT7 have been identified in various human cancers, including breast cancer, and are closely associated with tumor origin and progression 18. Moreover, SIRT7 expression has been found to be higher in metastasized tissue than in primary breast cancer tissue 19. Tang et al. reported that SIRT7 could be recruited for DNA damage repair at a later stage of DNA damage response, regulation of the dephosphorylation, inactivation of ataxia-telangiectasia mutant (ATM) through deacetylation to avoid continuous ATM activation, and assurance of the normal termination of DNA repair 20. Kiran et al. demonstrated that SIRT7 could reduce the DNA damage response by preventing the accumulation of γH2AX and p53, and inhibiting p38 and JNK activities 21. Additionally, SIRT7 can regulate the remodeling of chromatin structure and promote the modification of DNA double-strand breaks (DSBs) by catalyzing various histone modifications, such as H3K122 desuccinylation, H3K18 deacetylation, and H4K91 glutarylation 22-24. Given the crucial role of SIRT7 in maintaining genome stability and the close association between CIN and tumor metastasis, we hypothesized that SIRT7 dysregulation in breast cancer may potentiate metastasis by decreasing genomic stability. In this study, we tested the above hypothesis using cultured breast cancer cell lines, clinicopathological tissue samples, and mice models of breast cancer.

## Materials and Methods

### Cell culture

The breast cancer cell lines MCF-7 and SK-BR-3 were purchased from the Cell Bank of the Chinese Academy of Sciences (Shanghai, China). MCF-7 cells were cultured in minimum essential medium (MEM; Sigma, USA) supplemented with 10 % fetal bovine serum (FBS), 2 nm glutamine (Thermo Fisher Scientific, USA), and 5 μg/mL insulin (Beyotime, China). SK-BR-3 cells and neural crest stem cells (NCSCs) were cultured in dulbecco's modified eagle medium (DMEM) supplemented with 10% FBS and 2 nm glutamine (Thermo Fisher Scientific, USA).

### Patient tissue specimens

This study was approved by the Committee on Ethics of Shenzhen Second People's Hospital, Shenzhen University, Shenzhen, Guangdong, China. After obtaining written informed consent, tissue samples from 65 patients with breast cancer were collected from Shenzhen Second People's Hospital, Shenzhen, Guangdong, China, between January 2018 and December 2019. The expression levels of SIRT7 and LAP2α in the tumors were measured via immunohistochemistry.

### Western blotting

The cells were homogenized in a radioimmunoprecipitation assay (RIPA) buffer containing a protease inhibitor cocktail on ice for 30 min and subsequently centrifuged for 20 min at 12,000 rpm at 4°C. The loading buffer was added to the protein lysate, and the mixture was boiled for 5 min. The proteins were then separated on SDS polyacrylamide gels and transferred to polyvinylidene fluoride (PVDF) membranes (Millipore). The membrane was blocked with a triethanolamine-buffered saline solution (TBS) containing 5 % skim milk for 1 h and probed with the respective antibodies overnight. Next, the membrane was incubated with an alkaline phosphatase-conjugated secondary antibody for 2 h at room temperature. Finally, immunoreactions were detected using a 5-bromo-4-chloro-3-indolyl phosphate/Nitrotetrazolium Blue chloride (BCIP/NBT) substrate (Sangon Biotech, Shanghai, China). The following primary antibodies were used: SIRT7 (1:1000; cell signaling technology (CST), 5360S), LAP2α (1:1000; CST, 5369S), phospho-histone H2AX (γH2AX, 1:1000; CST, 9718S), and GAPDH (1:1000; CST, 2118S). The following secondary antibodies were used: anti-rabbit IgG horseradish peroxidase (HRP)-conjugated antibody (1:5000; CST, 7074S), and anti-rabbit IgG HRP-conjugated antibody (1:5000; CST, 7076S).

### Quantification of mRNA by real-time PCR

Total RNA was extracted using the PureLink RNA Mini Kit (Invitrogen, USA), and reverse transcription was conducted using a 5×PrimeScript RT Master Mix Kit (Takara, Japan). Quantitative real-time polymerase chain reaction (PCR) was performed using 2× TB Green Premix Ex Taq (Takara) to detect gene expression. Each gene's expression level was normalized to that of the housekeeping gene β-actin.

### Immunohistochemistry

Immunohistochemistry was used to detect SIRT7 and LAP2α expressions in breast cancer tissues. Five-micrometer-thick sections were sliced and deparaffinized, and antigens were retrieved in a microwave. Next, the slides were incubated in 3 % H_2_O_2_ deionized water at room temperature to block peroxidase. After protein blocking, the tissues were incubated with the indicated primary antibodies overnight at 4°C and then incubated with the biotinylated secondary antibody at room temperature for 30 min. Lastly, the slides were stained with diaminobenzidine for 10 min and counterstained with hematoxylin to label the nuclei. The following primary antibodies were used: SIRT7 (1:100; Signalway Antibody, 32106) and LAP2α (1:200; Abcam, ab226336).

### Gene knockdown and overexpression experiments

The SIRT7 gene silencing sequence was ligated into the retroviral pGFP-V-RS vector to construct a SIRT7 gene silencing short hairpin RNA (shRNA) lentiviral vector. The shRNA with the best interference efficiency against SIRT7 was screened before the corresponding lentivirus was packaged. The collected lentiviral vectors were then used to transfect MCF-7 and SK-BR-3 cells. Cells successfully transfected with the vector were screened using a medium containing puromycin. To transiently knockdown LAP2α expression, cells (1×10^5^) were transfected with LAP2α-specific small interfering RNA (siRNA) using Lipofectamine 2000 (Invitrogen). siRNAs were purchased from Shanghai GenePharma (Shanghai, China). The shRNA sequence for the SIRT7 gene was 5'-GCCAAATACTTGCTCGTCTAC-3'; the siRNA sequence for the SIRT7 gene was siRNA #1: 5'-GGAACAUGUACAUUGAAGUTT-3'; and the siRNA sequences for the LAP2α gene were siRNA #1: 5'-GCAGCUAAGAAAGUACAUATT-3'; siRNA#2: 5'-GGUCCUAUUGUGGGAACAATT-3'. The protein-coding gene was cloned into the pcDNA3.1(+) plasmid (Invitrogen) to express endogenous LAP2α in cells. Then, the cells were transfected with 150 nm of the recombinant plasmid using Lipofectamine 2000 (Invitrogen), and expression levels of exogenous proteins were assessed using western blotting.

### Co-immunoprecipitation (Co-IP) assay

After treatment, cells were harvested and lysed with IP lysis buffer (Meilunbio, China) containing a protease inhibitor cocktail on ice for 30 min, followed by centrifugation for 5 min at 12,000 rpm at 4°C. The antibodies against the target protein were incubated with Protein A/G-Agarose Beads (Invitrogen, USA) for 4 h at 4 °C, mixed with the lysate, and incubated at 4 °C for 16 h. After four washes with PBS, the Co-IP products were collected and analyzed by western blotting.

### Immunofluorescence microscopy

A total of 500 cells were seeded into a 20 mm glass-bottom cell culture dish and cultured for 1 day. The cells were washed with PBS and fixed in 4 % paraformaldehyde for 10 min. Subsequently, the cells were permeabilized with 1 % Triton X-100 for 5 min. The samples were blocked with 5 % BSA for 30 min and incubated with appropriate primary antibodies overnight. Next, the samples were washed with PBST three times, incubated for 2 h in the dark with the secondary antibodies coupled to Alexa Fluor, and stained the nuclei by incubating with 1 μg/mL DAPI (CST, 4083S) for 5 min. The primary antibodies used were as follows: SIRT7 (1:500; Abcam, ab62748), γH2AX (1:400; CST, 9718S), and LAP2α (1:500; Abcam ab5162); the secondary antibodies included Alexa Fluor 488 conjugated anti-mouse IgG (1:500, CST, 8878S) and Alexa Fluor 594 conjugated anti-rabbit IgG (1:500, CST, 8889S). Images were acquired using a fluorescence microscope.

### Wound healing and transwell invasion assays

MCF-7 or SK-BR-3 cells were cultured in six-well plates coated with 0.1 % gelatin for the wound healing assay. When 70 % confluence was reached, a wound was scratched at the center of the cell monolayer with a sterile plastic pipette tip and washed with PBS to remove the debris. Images were evaluated under a microscope 48 or 72 h after the scratch. Cell migration at the wound front was analyzed using an inverted microscope at the indicated times. For the transwell invasion assay, 1×10^5^ cells suspended in a medium without FBS were plated on the upper chamber (8-μm-pore carbonate membrane coated with matrigel, 6.5 mm insert, Corning). The insert was incubated in a 500 μL medium containing 10 % FBS. Invasive cells were stained with crystal violet, and non-invasive cells were removed by swiping the top of the membrane with cotton swabs.

### Animal studies

All animal studies were conducted with permission from the Committee on the Use of Live Animals in Teaching and Research at Shenzhen University. *In vivo* experiments were performed in BALB/c male nude mice aged 4-6 weeks with severe immune deficiency. After 3 days of adaptive feeding, the nude mice were randomly divided into four groups (control group, shSIRT7 group, siLAP2α, and shSIRT7/OELAP2α) with five mice in each group, and 1×10^5^ cells were inoculated into the tail vein of each mouse. The metastasis of tumor cells was monitored by *in vivo* imaging at 0, 7, 14, and 21 days after tumor cell injection, and five mice in each group were examined at each time point. After 21 days, the mice were sacrificed by cervical dislocation; their tumor sizes were measured with a caliper every 7 days, and the tumor volumes were calculated as (length × width × width)/2. The number of metastatic nodules on the lung surface was counted under a dissecting microscope after hematoxylin and eosin (HE) staining.

### Statistical analysis

All numerical data are presented as means ± standard deviation, and analyzed using one-way analysis of variance or Student's t-test. Differences were considered statistically significant at a p-value of <0.05.

## Results

### Doxorubicin inhibits SIRT7 expression and promotes breast cancer cell metastasis by enhancing CIN

**Figure 1:**
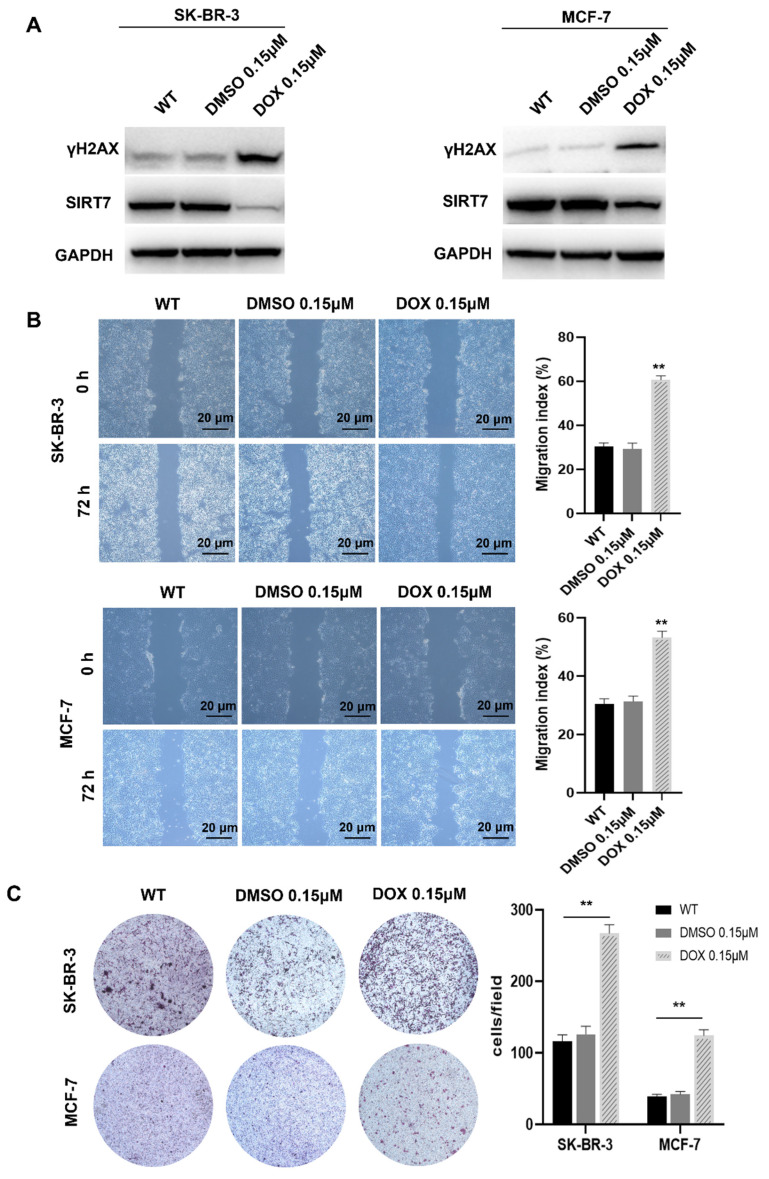
** Chromosomal instability suppresses SIRT7 expression and enhances the metastatic capacity of breast cancer cells. (A)** Western blot assay to detect the expression of γH2AX and SIRT7 protein in DOX-treated group and control group. **(B)** The wound-healing ability of the cells was detected by the scratch test; The quantification of each analysis is shown in the right figure. The experiments were carried out in triplicate (**p < 0.01). The scratch area was calculated using Image J software. Cell scratch area (0 hour) minus cell scratch area (72 hours) to get the cell migration area, the percentage of cell migration area to cell scratch area (0 hours) is the cell migration index. **(C)** Transwell assay to detect cell invasion ability; The quantification of each analysis is shown in the right figure. The experiments were carried out in triplicate (**p < 0.01). DOX: Doxorubicin.

Previous studies have shown that SIRT7 is a protective factor for cell genome stability and plays an important role in the metastasis of breast cancer cells 25, 26. To further clarify the relationship between SIRT7 and CIN in breast cancer cells, we treated SK-BR-3 and MCF-7 cells with the commonly used CIN inducer DOX 0.15 μM for 7 days 27. Western blotting was performed to detect the expression of γH2AX, a sensitive molecular marker of DNA damage and repair 28. Our data showed that the expression of γH2AX was significantly higher in the DOX treatment group than the control group. In contrast, the expression of SIRT7 was significantly downregulated in the DOX treatment group than in the control group **(Figure 1A)**. These data indicate that DOX treatment inhibited the SIRT7 expression and enhanced CIN in breast cancer cells.

CIN is closely associated with tumor cell metastasis 29, 30. To determine whether CIN plays the same role in breast cancer cells, we performed scratch and transwell assays to detect the metastatic ability of SK-BR-3 and MCF-7 cells after DOX treatment. Our data showed that in the scratch experiment, the wound healing rate of the cells treated with DOX was significantly higher than that of the control cells, and their migration ability was significantly enhanced **(Figure 1B)**. In the transwell experiment, DOX treatment increased the number of cells that passed through the bottom membrane of the inserts to more than twice that of the control group **(Figure 1C)**, suggesting that DOX treatment enhances the metastatic ability of breast cancer cells.

### SIRT7 deficiency leads to CIN in breast cancer cells

**Figure 2:**
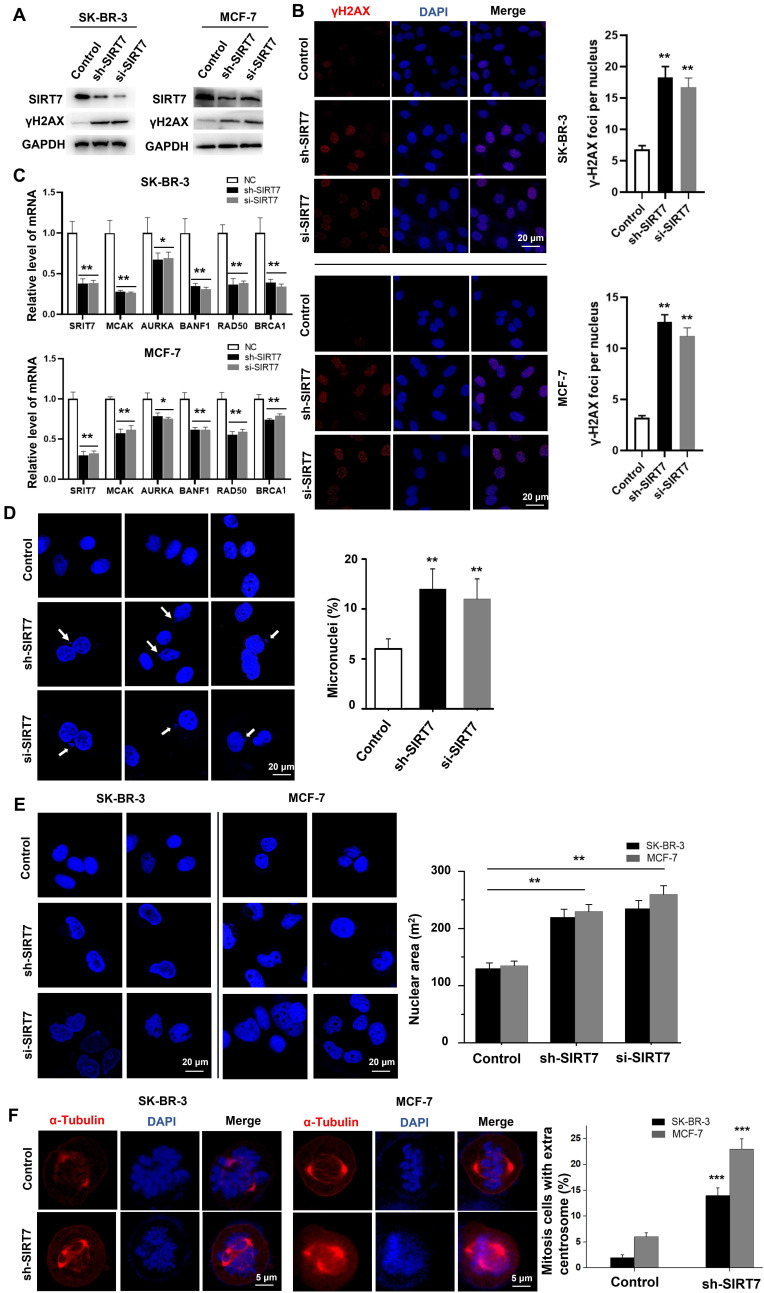
** Knockdown of SIRT7 results in chromosomal instability (CIN) in breast cancer cells. (A)** Western blot assay to detect the effect of knockdown of SIRT7 on the expression of DNA damage marker γH2AX in SK-BR-3 and MCF-7 cells. GAPDH was used as the control. **(B)** Immunofluorescence assay was used to detect the effect of knockdown of SIRT7 on the expression of γH2AX. Red is γH2AX staining, blue is DAPI staining of nuclei; the right panel counts the average number of lesions per nucleus (more than 40 cells in each group), The quantification of each analysis is shown in the right figure. The experiments were carried out in triplicate (**p < 0.01). **(C)** The expression level of chromosome stability-related genes between control and SIRT7-depleted cells was examined by quantitative real-time PCR. The experiments were carried out in triplicate (*p < 0.05; **p < 0.01). **(D)** Fluorescence confocal experiments showed micronuclei formation in the SIRT7 knockdown group (sh-SIRT7 and si-SIRT7) and control group in SK-BR-3 cells. The blue color is DAPI-stained nuclei, compared with the control group. The right histogram shows the percentage of micronucleus. The experiments were carried out in triplicate (**p < 0.01). **(E)** Fluorescence confocal experiments showed that the size and morphology of the nuclei in the knockdown group and control group of SK-BR-3 and MCF-7 cells were changed (more than 80 cells in each group). The right picture shows the quantitative analysis of the size of the nucleus using Fiji software. The experiments were carried out in triplicate (**p < 0.01). **(F)** Fluorescence confocal experiments showed the formation of spindles during mitosis in the SIRT7 knockdown group and control group in SK-BR-3 cells and MCF-7 cells (more than 80 cells in each group), red is α-tubulin-stained centrosomes and spindle filaments, blue DAPI-stained nuclei; the right panel shows the statistics of mitotic cells with multipolar spindles (more than 80 cells in each group), compared with the control group (*** p < 0.001).

To determine whether there is a causal link between CIN-induced SIRT7 downregulation and CIN-promoted CIN in breast cancer cells, we knocked down SIRT7 with shRNA and siRNA. We then assessed its effect on the expression of genome homeostasis- and chromosome stability-related genes in SK-BR-3 and MCF-7 breast cancer cells. Western blotting results showed that SIRT7 siRNA reduced the expression of SIRT7 by more than 90 % compared with control siRNA. SIRT7 knockdown significantly increased the expression of γH2AX compared with that of the control siRNA **(Figure 2A)**. Using immunofluorescence, we found that SIRT7 knockdown doubled the proportion of cells exhibiting γH2AX fluorescence in the nucleus. The average number of fluorescent foci per nucleus was significantly higher than that of control siRNA-treated cells **(Figure 2B)**. To further demonstrate the role of SIRT7 in regulating chromosome stability, we performed RT-qPCR to analyze the effect of SIRT7 knockdown on mRNA levels of five chromosome stability-related genes, namely mitotic centromere-associated kinesin (MCAK) 31, aurora kinase A (AURKA) 32, brrier-to-autointegration factor (BANF1) 33, RAD50 double strand break repair protein (RAD50) 34, and breast cancer type 1 susceptibility protein (BRCA1) 35. As shown in **Figure 2C**, these genes were significantly downregulated when SIRT7 was knocked down, especially MACK, BANF1, and RAD50.

Since CIN is mainly caused by the incorrect separation of chromosomes in the later stage of mitosis, leading to increased cytoplasmic micronuclei; therefore, we examined the incidence of micronuclei in SIRT7 knockdown cells. We counted the number of micronuclei in all cells (more than 80 cells per group), and a representative field was displayed. As shown in **Figure 2D**, there was a significantly higher occurrence rate of micronuclei in breast cancer cells with SIRT7 knockdown. In addition, we observed a change in nuclear morphology after SIRT7 knockdown, with an approximately 0.5-fold increase in the average area of the nuclei and more irregular shapes **(Figure 2E)**. We also performed fluorescence confocal microscopy experiments to examine the spindle formation of spindles during mitosis in SK-BR-3 cells and MCF-7 cells transfected with SIRT7 shRNA/siRNA or control siRNA by counting the centrosomes stained with α-tubulin during mitosis and the ratio of spindle filaments to the whole cell. More than 80 cells were counted for each treatment. We found that the frequency of cells with multipolar spindles was significantly higher in the SIRT7 knockdown group and that the formation of multipolar spindles caused abnormal telophase chromatin segregation **(Figure 2F)**.

These results indicate that SIRT7 deficiency promotes CIN in breast cancer cells.

### SIRT7 interacts directly and positively correlates with LAP2α in breast cancer

**Figure 3:**
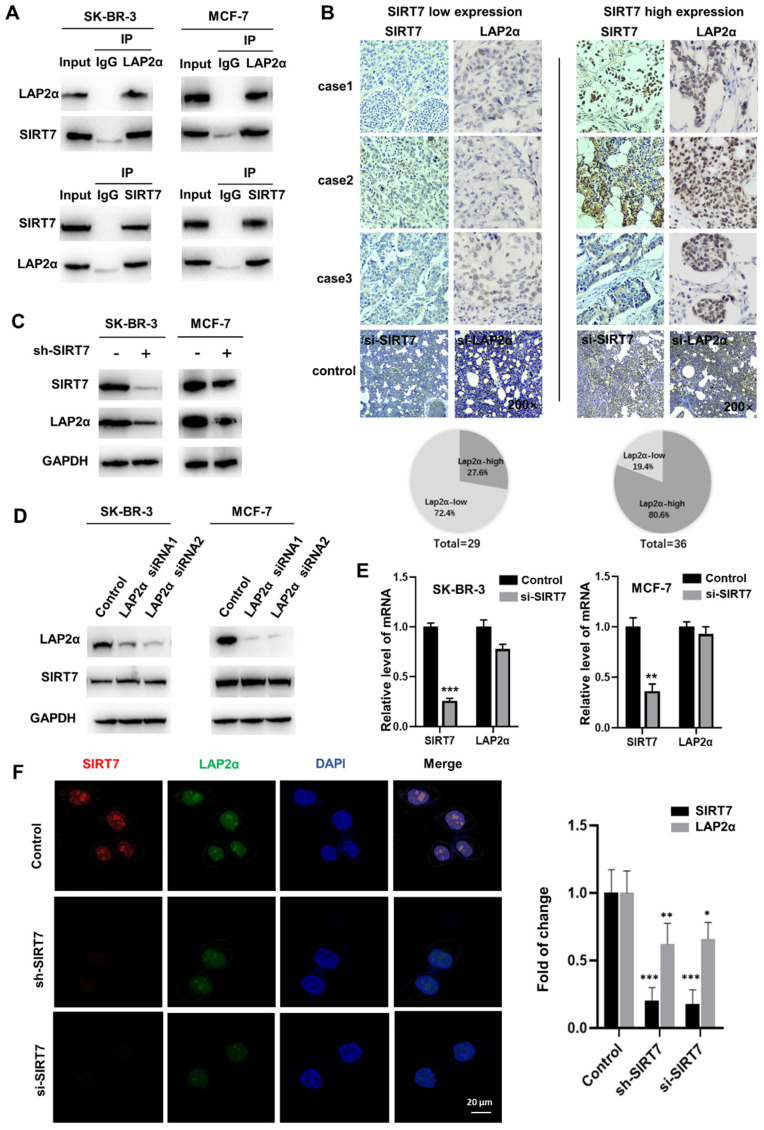
** SIRT7 interacts and positively correlates with LAP2α in breast cancer. (A)** Co-IP assays showing SIRT7 binding to LAP2α in breast cancer cells. **(B)** Detection expression levels of SIRT7 and LAP2α in breast cancer pathological tissue by immunohistochemistry. Proportion analysis of LAP2α expression in SIRT7 high and low expression groups. SIRT7 knockdown group and LAP2α knockdown group was used as control. **(C)** Western blot assay was used to detect the expression levels of SIRT7 and LAP2α in SK-BR-3 and MCF-7 cells in the SIRT7 knockdown group and control group. **(D)** Western blot assay was used to detect the expression levels of SIRT7 and LAP2α in SK-BR-3 and MCF-7 cells in the LAP2α knockdown group and control group. **(E)** The expression level of LAP2α in SK-BR-3 and MCF-7 cells was measured by quantitative real-time PCR. The experiments were carried out in triplicate (**p < 0.01; ***p < 0.001). **(F)** Immunofluorescence assay to detect the fluorescence expression and localization of SIRT7 and LAP2α in the SIRT7 knockdown group and control group of SK-BR-3 cells, red is SIRT7 staining, green is LAP2α protein, blue is DAPI-stained nuclei; The right graph counts the average fluorescence intensity of each protein index. The experiments were carried out in triplicate (*p < 0.05; **p < 0.01; ***p < 0.001).

To identify the downstream targets of SIRT7 in regulating cell chromosome stability, we incubated an anti-SIRT7 antibody with thermo scientific pierce A/G magnetic beads, and the pulled-down protein gel strips that were bound to SIRT7 protein were subjected to mass spectrometry analysis. Co-IP experiments showed that the SIRT7 antibody could pull down LAP2α protein in different breast cancer cells **(Figure 3A)**. To further determine the interaction between SIRT7 and LAP2α, we analyzed the expression of these two proteins in cancerous tissues collected from 65 patients with breast cancer who underwent surgical resection. We divided the tissues into two groups, SIRT7 low and high expression groups, according to the expression level of SIRT7, as assessed by immunohistochemistry. **Figure 3B** shows the low expression level of LAP2α (72.4%) in the SIRT7 low expression samples, and the high expression level of LAP2α (80.6%) in the SIRT7 high expression samples.

To study the regulatory relationship between SIRT7 and LAP2α, we investigated the interaction between SIRT7 and membrane-associated protein LAP2α. We performed western blotting and found that SIRT7 knockdown with siRNA significantly decreased the expression of LAP2α in SK-BR-3 and MCF-7 cells** (Figure 3C)**. Notably, LAP2α knockdown did not affect the expression level of SIRT7 **(Figure 3D)**. Then, we performed RT-qPCR to detect mRNA expression in cells transfected with SIRT7 siRNA or control siRNA and found that the mRNA expression of LAP2α was unaffected by either siRNAs **(Figure 3E)**. Using fluorescence staining, we observed a co-localization of LAP2α and SIRT7 in the nuclei of the cells. Compared with control siRNA, SIRT7 siRNA led to more than a 50 % reduction in the cell's mean fluorescence intensity of LAP2α **(Figure 3F)**.

These results indicate that SIRT7 directly interacts with and positively correlates with the LAP2α protein in breast cancer cells.

### SIRT7 knockdown promotes ubiquitination and degradation of LAP2α in breast cancer cells

**Figure 4:**
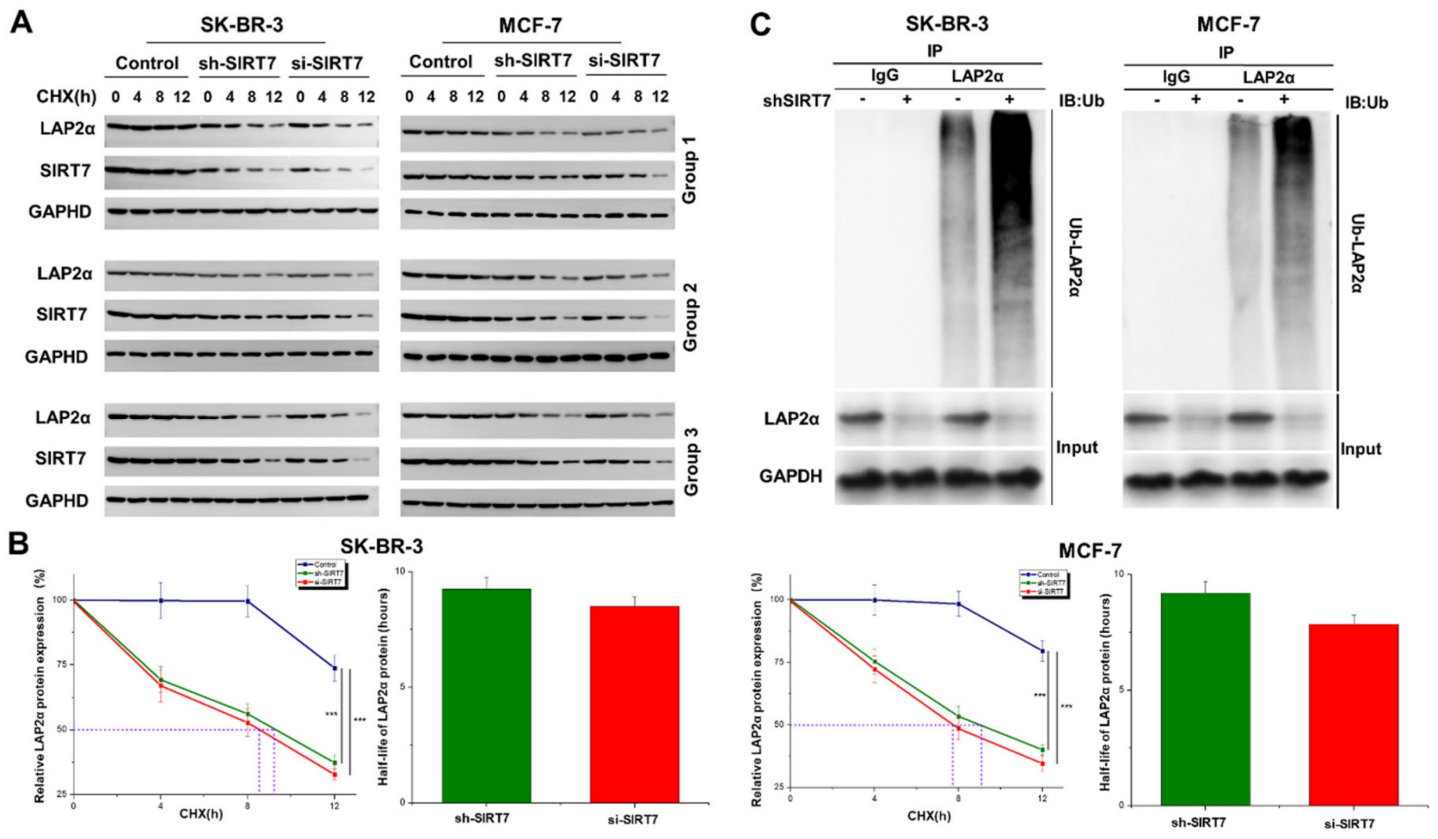
** SIRT7 inhibits ubiquitination-mediated degradation of LAP2α. (A)** Western blot analysis of the protein degradation level of LAP2α in SK-BR-3 and MCF-7 cells. **(B)** The half-life of LAP2α in SK-BR-3 and MCF-7 cells. The experiments were carried out in triplicate (***p < 0.001). **(C)** Detection of ubiquitination levels of LAP2α protein using an anti-ubiquitin antibody.

We further explored the molecular mechanism of SIRT7-mediated regulation of chromosome stability. Based on the above results that SIRT7 may regulate the expression of LAP2α through protein interactions, we speculated that SIRT7 knockdown promotes the degradation of LAP2α protein in breast cancer cells. We used the protein synthesis inhibitor cycloheximide (CHX, 50 μg/mL) to treat SK-BR-3 and MCF-7 cells transfected with SIRT7 siRNA or control siRNA at different time points. The western blot results showed that in SIRT7 siRNA-treated cells, the degradation of LAP2α protein was accelerated, and the protein level of LAP2α gradually decreased **(Figure 4A)**. The quantified half-life of LAP2α is shown in **Figure 4B**. To further determine whether LAP2α degradation was ubiquitination-dependent, we measured the levels of intracellular ubiquitin-proteins using an IP assay. As shown in **Figure 4C**, SIRT7 knockdown significantly increased the ubiquitination level of LAP2α. These results suggest that SIRT7 knockdown promotes the ubiquitination and degradation of LAP2α in breast cancer cells.

### SIRT7 regulates CIN through LAP2α in breast cancer cells

We further investigated the relationship between SIRT7, LAP2α, and CIN in breast cancer. RT-qPCR results showed that LAP2α knockdown decreased the expression levels of chromosome stability-related genes, including ATM 36, MCAK, AURKA, BANF1, RAD50, BRCA1, and BRCA2.

**Figure 5:**
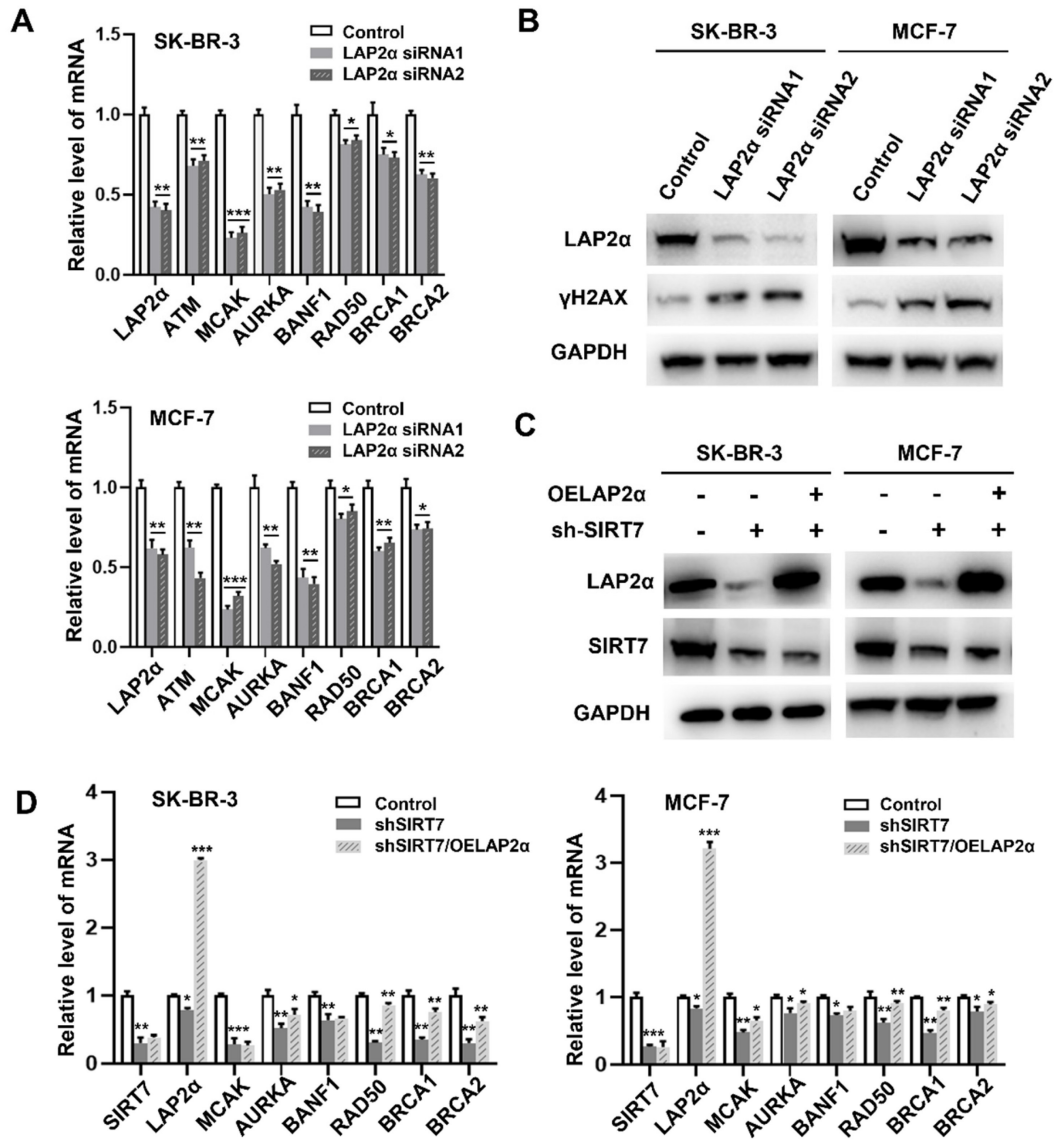
** SIRT7 regulates chromosome instability through LAP2α. (A)** Effects of LAP2α knockdown on the expression level of chromosome stability-related genes. RT-qPCR was used to evaluate the expression level. The experiments were carried out in triplicate. (*p < 0.05; **p < 0.01; ***p < 0.001).** (B)** The expression of γH2AX was detected by western blotting in control and siLAP2α cells.** (C)** Immunoblots showing LAP2α levels in cells expressing sh-SIRT7 or overexpressing LAP2α.** (D)** Impact of overexpression LAP2α on chromosome stability-related genes in SIRT7 knockdown breast cells. The expression levels were examined by RT-qPCR. The sh-SIRT7 cells compared with control cells, overexpressing LAP2α cells compared with sh-SIRT7 cells. The experiments were carried out in triplicate. (*p < 0.05; ** p < 0.01; *** p < 0.001).

MACK and BANF1 levels were the most significantly reduced **(Figure 5A)**. LAP2α knockdown was confirmed by western blotting **(Figure 5B)**. Notably, the expression level of γH2AX was significantly higher than that in cells transfected with control siRNA. Therefore, we investigated whether SIRT7 could affect CIN by regulating the expression of LAP2α. To test this possibility, we overexpressed LAP2α in SK-BR-3 and MCF-7 cells, which were knocked down by siRNA. Our data showed that SIRT7 was significantly knocked down, and its expression was reduced by approximately one-third of the original **(Figure 5C)**. We also detected the mRNA levels of genes related to chromosome stability by RT-qPCR in the SIRT7 knockdown, LAP2α overexpression, and control groups, respectively. The results showed that when SIRT7 is knocked down, the expression of chromosome stability-related genes, such as MACK, RAD50, and BRCA1, is significantly compensated for by LAP2α overexpression **(Figure 5D)**.

These results indicate that SIRT7 downregulation promotes CIN by suppressing the expression of LAP2α in breast cancer cells.

### SIRT7 knockdown promotes breast cancer cell metastasis by downregulating LAP2α

**Figure 6:**
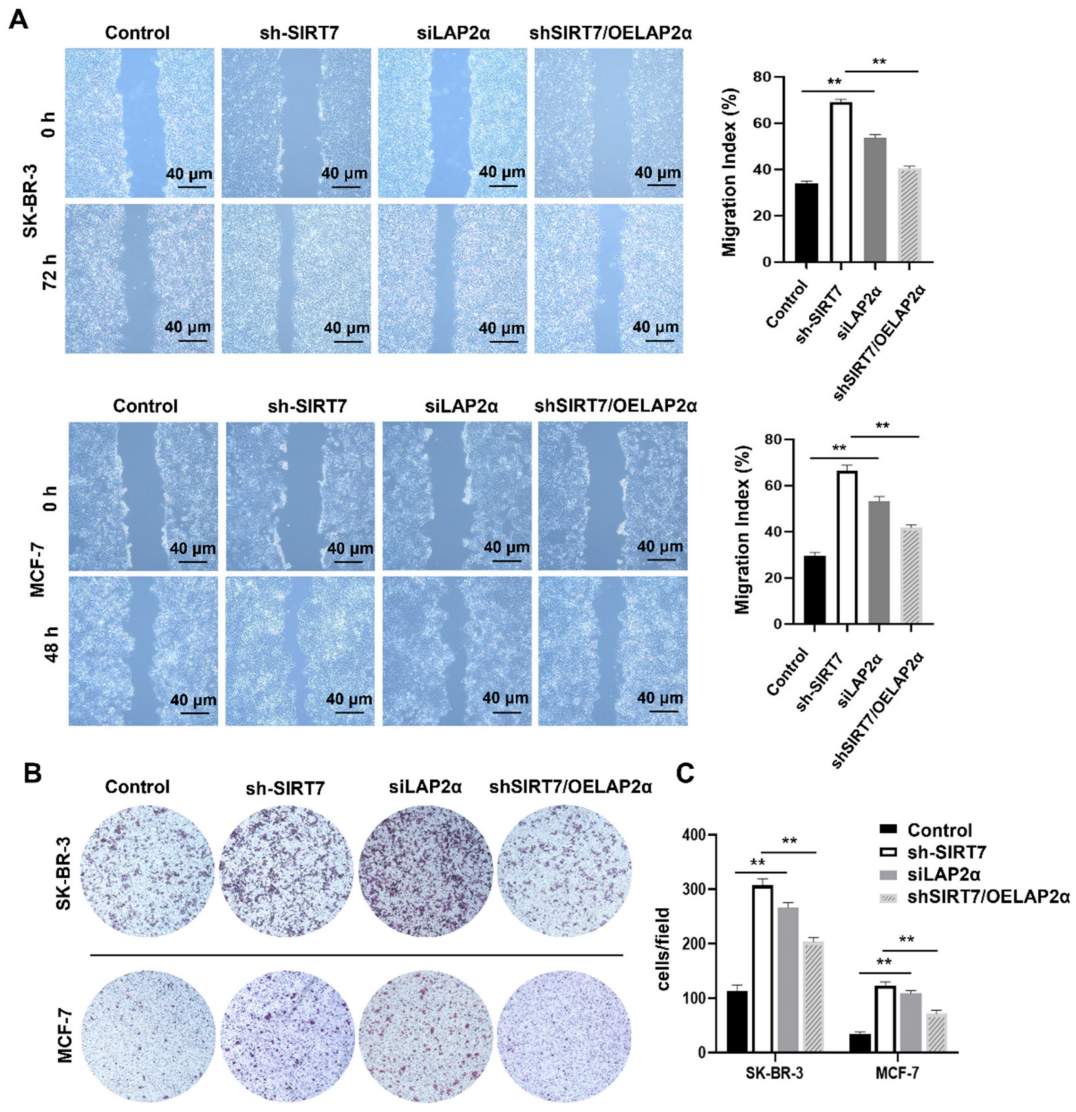
**Knockdown of SIRT7 promotes breast cancer cell metastasis by downregulating LAP2α. (A)** Wound healing assay of SIRT7 or LAP2α knockdown separately and LAP2α-overexpressing breast cancer cells. The quantification of each analysis is shown in the right figure. The experiments were carried out in triplicate (**p < 0.01). **(B)** The effect of SIRT7 and LAP2α on cell invasion capability was measured by the transwell migration assay. **(C)** Invasion of cells by transwell invasion assay. The quantification of each analysis is shown in the right figure. The experiments were carried out in triplicate (**p < 0.01).

To investigate whether SIRT7 promotes breast cancer metastasis through the SIRT7/LAP2α axis, we assessed cell migration changes after the knockdown or overexpression of SIRT7 and LAP2α using scratch and transwell assays, respectively. When SIRT7 or LAP2α was knocked down, scratch wound healing and transwell assays showed increased migration and invasion capabilities of SK-BR-3 and MCF-7 cells. The closure of the scratch gap in SIRT7- or LAP2α- knockdown cells healed significantly faster than that in the control group. However, no significant difference was reported in scratch healing between the SIRT7-knockdown plus LAP2α-overexpression and control groups **(Figure 6A)**. Similarly, in the transwell experiments, the number of invading cells in the SIRT7- or LAP2α- knockdown group was significantly increased, approximately three times more than that in the control group; However, when LAP2α was overexpressed in SIRT7- knockdown cells, the acquired enhancement in migration and invasion was lost **(Figure 6B, C)**.

**Figure 7:**
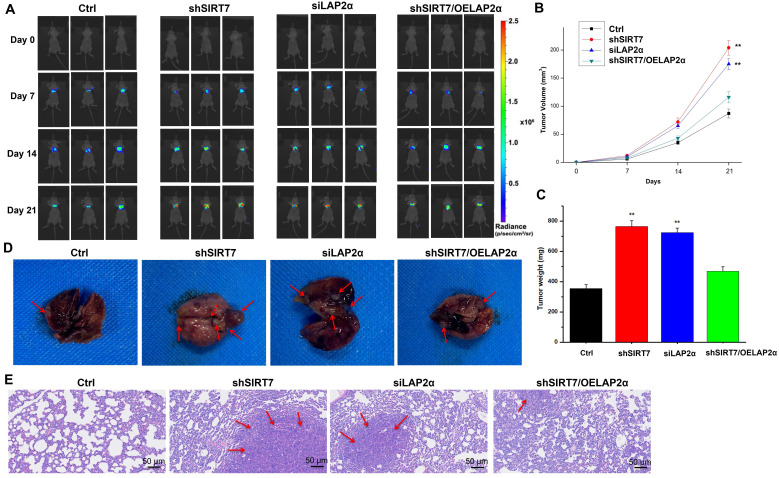
** Knockdown of SIRT7 promotes breast cancer metastasis *in vivo*. (A)** The metastatic ability of MCF-7/ADR cells was assessed by injecting 1×10^6^ SIRT7 or LAP2α knockdown separately and LAP2α-overexpressing breast cancer cells into the lateral tail vein of nude mice. Luciferase activity in each mouse was measured using an *in vivo* imaging system. **(B, C)** The tumor volumes and weight in the mice were measured every 7 days. The mean of each group is indicated. The experiments were carried out in triplicate (**p < 0.01), 21 days later, the mice were euthanized. **(D)** The number of metastatic nodules on the surface of the lungs.** (E)** Hematoxylin-eosin (HE)-stained sections showed lung tissue of mice. The red arrows point at cell invasion areas.

Finally, to determine whether the SIRT7/LAP2α axis plays a role in breast cancer metastasis *in vivo*, we injected SIRT7-knockdown, LAP2α-knockdown, or SIRT7-knockdown plus LAP2α-overexpression SK-BR-3 cells into the tail vein of athymic nude mice to achieve systemic dissemination, followed by tracking of metastatic colonization using a bioluminescent reporter. Our data showed that SIRT7-knockdown and LAP2α-knockdown cells were more susceptible to metastasis than control cells **(Figure 7A)**. By measuring the changes in tumor volume over time, we found that the tumor volumes of both SIRT7-knockdown and LAP2α-knockdown groups were increased approximately twice than that of the control group **(Figure 7B)**. The tumor weights of these two groups were also significantly increased, approximately twice than that of the control group **(Figure 7C)**. However, in SIRT7-knockdown plus LAP2α-overexpression group, we observed a slightly greater increase in the tumor volume and weight than in the control group. Moreover, the mice injected with SIRT7-knockdown, and LAP2α-knockdown cells showed fewer lung metastatic nodules than the control cells **(Figure 7D)**. Consistent with the above results, HE-stained sections showed more significant cell invasion in the lungs of the mice injected with SIRT7-knockdown and LAP2α-knockdown cells than in the control cells **(Figure 7E)**. These *in vivo* results indicate that the SIRT7 knockdown can promote the metastasis of breast cancer cells, and LAP2α overexpression can suppress this increased metastatic potential.

## Discussion

Tumor metastasis is the main factor affecting the quality of life and survival of patients with breast cancer 37. Therefore, studying the metastatic mechanism of breast cancer is of immense significance for improving the therapeutic effects of breast cancer. CIN is an important driver of tumorigenesis and progression, an important hallmark of human cancer and promotes tumor metastasis in cancer patients 38-41. However, the molecular mechanisms underlying CIN-driven tumor metastasis still need to be elucidated. In the present study, our data demonstrated that DOX treatment inhibited SIRT7 expression and enhanced CIN and metastasis in breast cancer cells. Mechanistically, we established a causal link between SIRT7 downregulation and enhanced CIN in DOX-treated cells, in which SIRT7 interacted directly with LAP2α to promote its ubiquitination and degradation and to regulate CIN and metastasis of breast cancer cells. Our data suggest an important role of the SITR7/LAP2α pathway in CIN- induced metastasis in breast cancer.

As a chromatin regulatory protein, SIRT7 has various enzymatic activities and plays an important role in remodeling the chromosome structure, DNA damage repair, regulation of the mitotic cycle, and regulation of transcription factors 42-44. Abnormal DNA damage repair is an important factor in the formation of CIN. A continuous increase or abnormal repair of DNA damage may lead to abnormal separation of chromosomes during mitosis; furthermore, severe DNA may even lead to fragmentation of chromosome fragments 45. Here, our data showed that SIRT7 knockdown promoted CIN in breast cancer cells, as evidenced by compromised DNA repair capacity, increased number of cytoplasmic micronuclei, increased formation of multipolar spindles and downregulated expression of genes that maintain chromatin stability. The morphological heterogeneity of nuclei is the gold standard for the clinicopathological diagnosis and grading of cancer; for example, changes in the size, shape, and number of nucleoli can be used to determine the malignancy of tumor cells 46. In this study, we found that the expression of γH2AX, a marker of CIN, was significantly increased in the DOX treatment group, which was accompanied by the downregulated expression of SIRT7, simultaneous indication inhibits of SIRT7 expression and enhancement of CIN in breast cancer cells. Additionally, our data demonstrated that DOX promoted the metastatic ability of breast cancer cells, implying exploration of the relationship between SIRT7 downregulation and enhanced CIN and metastasis in breast cancer cells. Indeed, when we knocked down SIRT7 in breast cancer cells, abnormal changes in nuclear size and morphology that favored CIN were observed. These data clearly indicate that SIRT7 knockdown affects chromosome stability in multiple ways, suggesting that SIRT7 plays an important role in regulating chromatin stability in breast cancer cells.

We next explored the potential mechanism by which SIRT7 knockdown contributed to CIN in breast cancer cells and identified the involvement of LAP2α protein. Previous studies have shown that LAP2α is involved in maintaining the structural stability of chromosomes. A lack of LAP2α can cause instability of the nuclear envelope and chromosomes during mitosis, causing CIN 47. Notably, loss of LAP2α may directly impair the assembly of the inner nuclear envelope, which maintains the normal structure of the nuclear envelope 48. As an important structure separating chromatin and the cytoplasm, the nuclear membrane also plays an important role in maintaining the normal structure of chromatin 49. The inner nuclear membrane can provide an attachment site in the nucleus for peripheral chromosomes and participates in DNA replication, transcription, and other biological processes 50, 51. Recently, an increasing number of studies have shown that the interaction between chromatin, histone-modifying enzymes, and the inner nuclear membrane is important for regulating the structure and function of chromatin 52. We used several common genes that regulate chromosome stability to verify that SIRT7 and LAP2α knockdown leads to chromosome instability. Impaired ATM function affects G1/S checkpoint switching, resulting in the unlimited replication of damaged DNA and genomic instability 36. The maladjustment of MCAK leads to defects in spindle assembly, chromosome aggregation and separation in mammalian cells, further leading to chromosome instability 31, 53. AURKA and BANF1 are associated with centrosome amplification, mitotic abnormalities, and chromosome instability 32, 33. DNA repair protein RAD50 expression causes cell instability, increasing the risk of DNA damage accumulation and malignant transformation 34. Lose breast cancer type 1/2 susceptibility protein (BRCA1/2) activity causes genomic instability and chromosomal rearrangements 35. These genes were significantly downregulated when SIRT7 or LAP2α were knocked down, especially MACK and BANF1. These results indicate that SIRT7 and LAP2α knockout cause chromosome instability. LAP2α maintains genomic integrity by aiding RPA deposit on damaged chromatin 54, and this result is consistent with the experimental results. Moreover, our results showed that SIRT7 regulates chromatin stabilization by directly binding to LAP2α protein in cancer cells. Our data support that SIRT7 acts as an upstream molecule of LAP2α, and SIRT7 knockdown causes downregulation of LAP2α expression in breast cancer.

Furthermore, our Co-IP results indicated a direct interaction between SIRT7 and LAP2α in breast cancer cells, in which SIRT7 knockdown increases the ubiquitination and degradation of LAP2α protein and consequently leads to a decreased protein level of LAP2α. In addition, we observed a positive correlation between SIRT7 and LAP2α expression in clinical breast cancer samples. Collectively, these results suggest that SIRT7 contributes to the maintenance of chromosomal stability by inhibiting LAP2α degradation, which may also explain the molecular mechanism by which SIRT7 knockdown leads to CIN.

This study has a few limitations. Although our data demonstrated that SIRT7 regulates effect on LAP2α, we did not design experiments to clarify whether SIRT7 inhibits LAP2α degradation through its enzymatic function. Moreover, it remains unclear whether the binding of SIRT7 to LAP2α could cause changes in the conformation of the LAP2α protein and subsequently affect the normal structure or function of these complexes, or whether SIRT7 regulates the function of these complexes by acting as a histone deacetylase that affects chromosomal histones and DNA chains. Finally, the tightness of binding between SIRT7 and LAP2α, which in turn affects the binding of these chromatin regulatory protein complexes to chromatin, remains a question to be answered in future studies.

**Figure 8:**
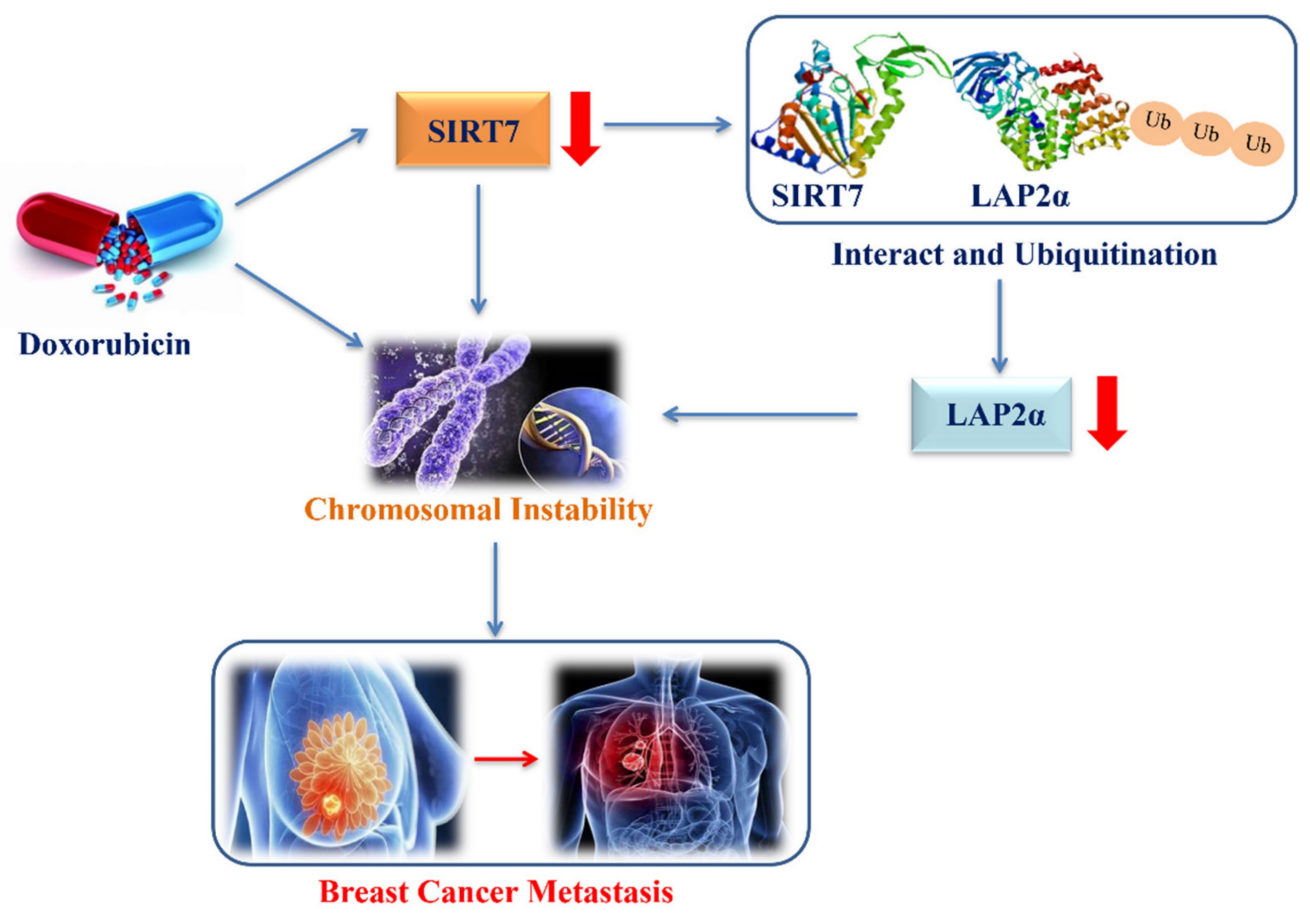
The proposed model of down-regulation of SIRT7 promotes breast cancer metastasis via LAP2α -induced chromosomal instability.

Previous studies have shown that SIRT7 plays a dual role in tumor development. On the one hand, research evidence shows that high expression levels of SIRT7 are associated with aggressive cancer phenotypes, tumor metastases, and poor patient outcomes in epithelial prostate and ovarian cancers 55, 56. On the other hand, studies have reported that the downregulation of SIRT7 expression promotes metastatic tumors, and the knockdown of SIRT7 increases the epithelial-mesenchymal transition of breast cancer cells by upregulating the TGF-β pathway, which further leads to an increase in breast cancer cell metastasis 18. This paradoxical role in tumorigenesis and development may be attributed to the complex molecular mechanisms by which SIRT7 regulates different types of cancer. Although the importance of CIN in promoting tumor evolution has been well documented, the exact molecular mechanisms by which CIN drives tumor development still require further exploration. Here, our data revealed that SIRT7 may interact with LAP2α to promote CIN in breast cancer cells, which is supported by our finding that the decreased low transcription levels of chromosomal stability-related genes induced by SIRT7 knockdown were partially compensated for by LAP2α complementation in cancer cells. Furthermore, our data demonstrate an enhanced metastatic phenotype of breast cancer metastasis cells when LAP2α protein is knocked down and degraded due to SIRT7 knockdown, and LAP2α overexpression significantly reversed the change in the metastatic phenotype induced by SIRT7 knockout. These findings suggest a new mechanism by which SIRT7 downregulation promotes breast cancer metastasis via LAP2α-induced chromosomal instability** (Figure 8)**.

In summary, our work demonstrates the important role of the SITR7/LAP2α pathway in promoting CIN-induced metastasis in breast cancer and that the SITR7/LAP2α pathway may represent a new therapeutic approach in preventing the metastasis of breast cancer cells. However, further studies are needed to clarify the molecular mechanism by which SIRT7 in regulates chromosomal stability and breast cancer cell metastasis.

## Abbreviations

CIN: Chromosomal instability
LAP: lamina-associated polypeptide
TRF1: telomere repeat binding factor 1
WRN: werner syndrome helicase
Rb: retinoblastoma protein
ATM: ataxia-telangiectasia mutant
NCSCs: neural crest stem cells
DMEM: dulbecco's modified eagle medium
RIPA: radioimmunoprecipitation assay
PVDF: polyvinylidene fluoride
TBS: triethanolamine-buffered saline solution
BCIP/NBT: 5-bromo-4-chloro-3-indolyl phosphate/Nitrotetrazolium Blue chloride
CST: cell signaling technology
HRP: horseradish peroxidase
HE: hematoxylin and eosin
DSB: double-strand breaks
DOX: doxorubicin
FBS: fetal bovine serum
RIPA: radioimmunoprecipitation assay
PCR: polymerase chain reaction
shRNA: short hairpin RNA
CHX: cycloheximide
siRNA: small interfering RNA
MCAK: mitotic centromere-associated kinesin
AURKA: aurora kinase A
BANF: brrier-to-autointegration factor
BRCA1/2: breast cancer type 1/2 susceptibility protein
